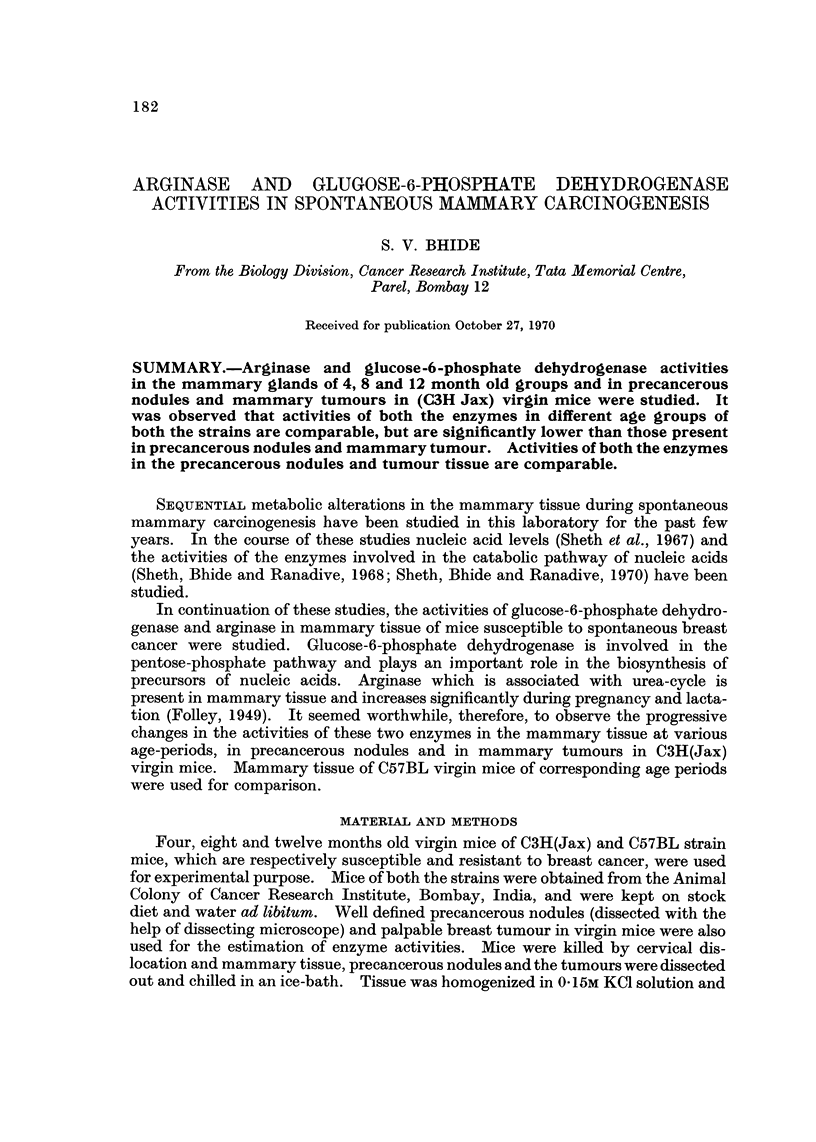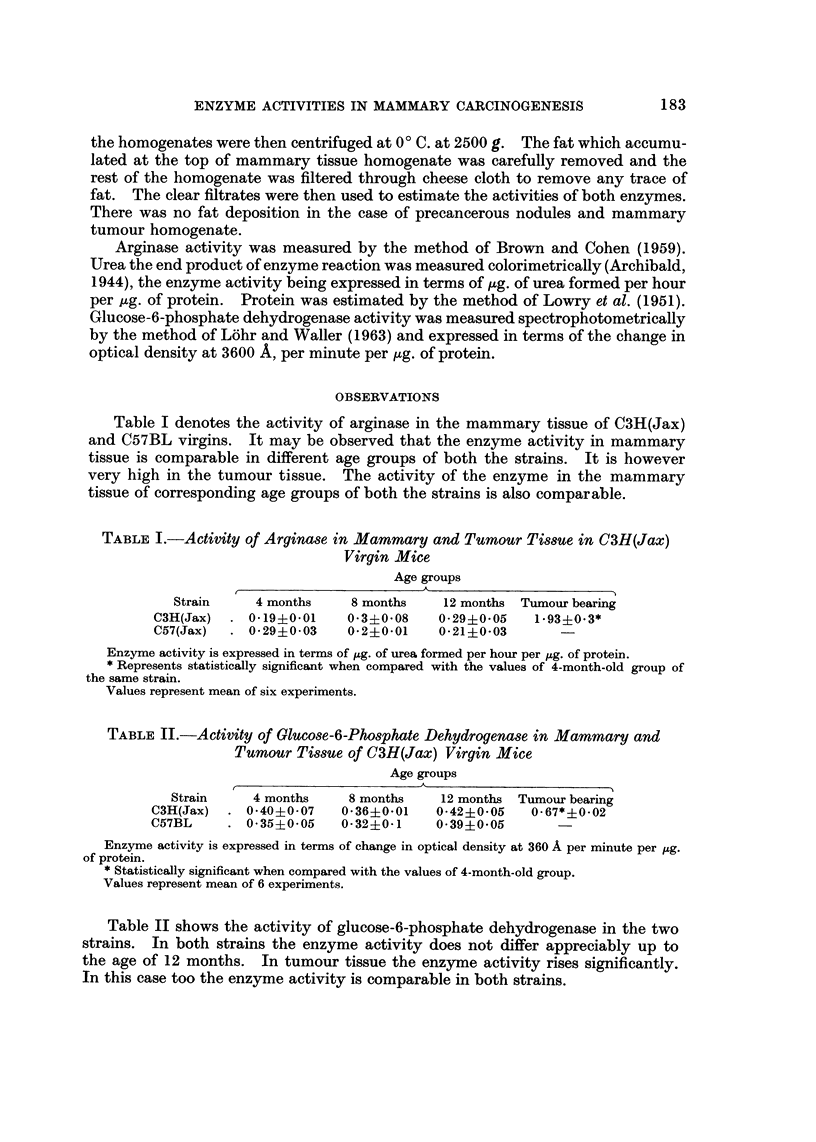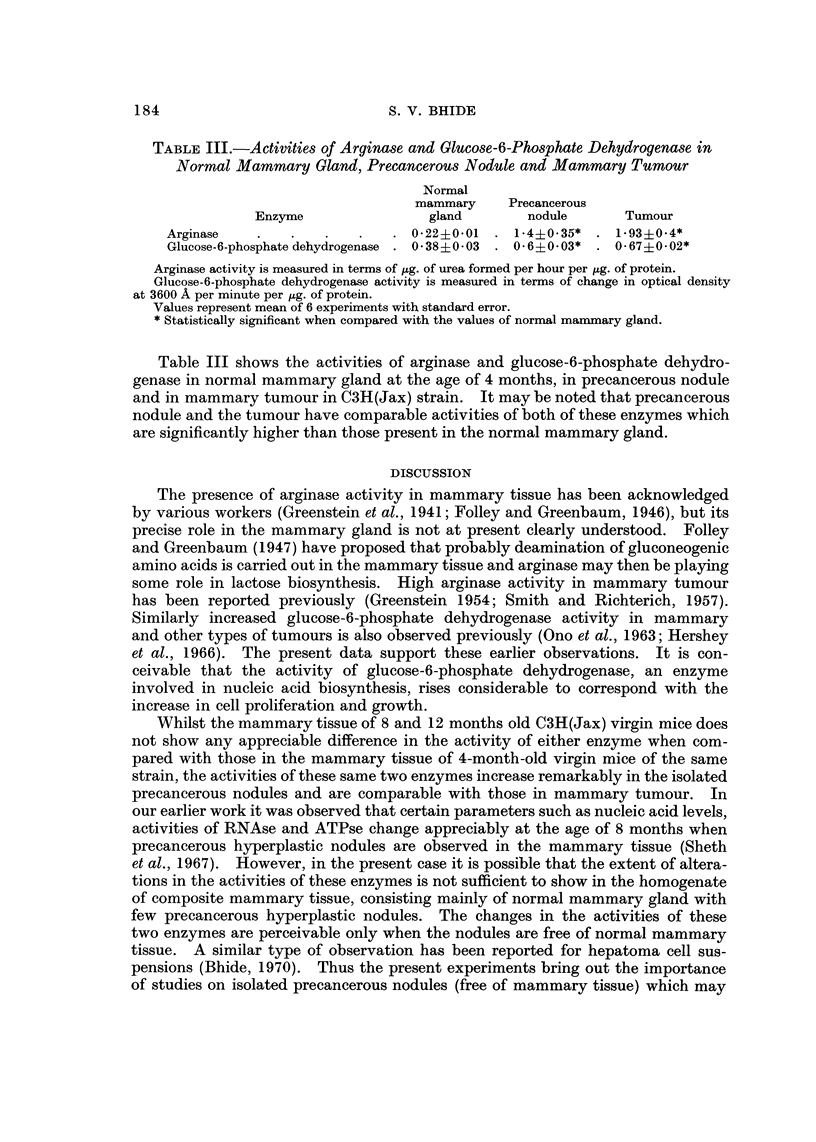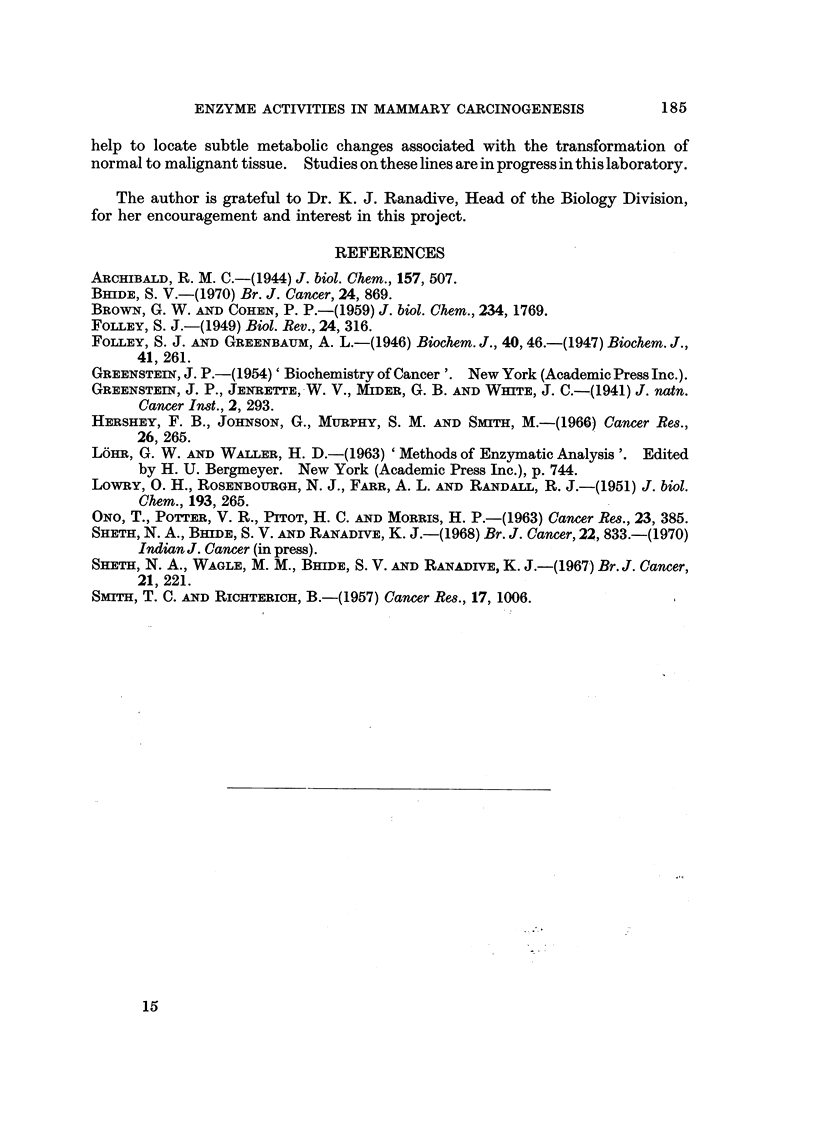# Arginase and Glugose-6-Phosphate Dehydrogenase Activities in Spontaneous Mammary Carcinogenesis

**DOI:** 10.1038/bjc.1971.25

**Published:** 1971-03

**Authors:** S. V. Bhide

## Abstract

Arginase and glucose-6-phosphate dehydrogenase activities in the mammary glands of 4, 8 and 12 month old groups and in precancerous nodules and mammary tumours in (C3H Jax) virgin mice were studied. It was observed that activities of both the enzymes in different age groups of both the strains are comparable, but are significantly lower than those present in precancerous nodules and mammary tumour. Activities of both the enzymes in the precancerous nodules and tumour tissue are comparable.


### ARGINASE AND GLUGOSE-6-PHOSPHATE DEHYDROGENASE ACTIVITIES IN SPONTANEOUS MAMMARY CARCINOGENESIS

S. V. BHIDE

*From the Biology Division, Cancer Research Institute, Tata Memorial Centre, Parel, Bombay* 12

Received for publication October 27, 1970

**SUMMARY.—Arginase and glucose-6-phosphate dehydrogenase activities in the mammary glands of 4, 8 and 12 month old groups and in precancerous nodules and mammary tumours in (C3H Jax) virgin mice were studied. It was observed that activities of both the enzymes in different age groups of both the strains are comparable, but are significantly lower than those present in precancerous nodules and mammary tumour. Activities of both the enzymes in the precancerous nodules and tumour tissue are comparable.**

SEQUENTIAL metabolic alterations in the mammary tissue during spontaneous mammary carcinogenesis have been studied in this laboratory for the past few years. In the course of these studies nucleic acid levels (Sheth *et al.*, 1967) and the activities of the enzymes involved in the catabolic pathway of nucleic acids (Sheth, Bhide and Ranadive, 1968; Sheth, Bhide and Ranadive, 1970) have been studied.

In continuation of these studies, the activities of glucose-6-phosphate dehydrogenase and arginase in mammary tissue of mice susceptible to spontaneous breast cancer were studied. Glucose-6-phosphate dehydrogenase is involved in the pentose-phosphate pathway and plays an important role in the biosynthesis of precursors of nucleic acids. Arginase which is associated with urea-cycle is present in mammary tissue and increases significantly during pregnancy and lactation (Folley, 1949). It seemed worthwhile, therefore, to observe the progressive changes in the activities of these two enzymes in the mammary tissue at various age-periods, in precancerous nodules and in mammary tumours in C3H(Jax) virgin mice. Mammary tissue of C57BL virgin mice of corresponding age periods were used for comparison.

### MATERIAL AND METHODS

Four, eight and twelve months old virgin mice of C3H(Jax) and C57BL strain mice, which are respectively susceptible and resistant to breast cancer, were used for experimental purpose. Mice of both the strains were obtained from the Animal Colony of Cancer Research Institute, Bombay, India, and were kept on stock diet and water *ad libitum*. Well defined precancerous nodules (dissected with the help of dissecting microscope) and palpable breast tumour in virgin mice were also used for the estimation of enzyme activities. Mice were killed by cervical dislocation and mammary tissue, precancerous nodules and the tumours were dissected out and chilled in an ice-bath. Tissue was homogenized in 0·15M KCl solution and the homogenates were then centrifuged at 0° C. at 2500 *g*. The fat which accumulated at the top of mammary tissue homogenate was carefully removed and the rest of the homogenate was filtered through cheese cloth to remove any trace of fat. The clear filtrates were then used to estimate the activities of both enzymes. There was no fat deposition in the case of precancerous nodules and mammary tumour homogenate.

Arginase activity was measured by the method of Brown and Cohen (1959). Urea the end product of enzyme reaction was measured colorimetrically (Archibald, 1944), the enzyme activity being expressed in terms of μg. of urea formed per hour per μg. of protein. Protein was estimated by the method of Lowry *et al.* (1951). Glucose-6-phosphate dehydrogenase activity was measured spectrophotometrically by the method of Löhr and Waller (1963) and expressed in terms of the change in optical density at 3600 Å, per minute per μg. of protein.

### OBSERVATIONS

Table I denotes the activity of arginase in the mammary tissue of C3H(Jax) and C57BL virgins. It may be observed that the enzyme activity in mammary tissue is comparable in different age groups of both the strains. It is however very high in the tumour tissue. The activity of the enzyme in the mammary tissue of corresponding age groups of both the strains is also comparable.

TABLE I.—*Activity of Arginase in Mammary and Tumour Tissue in C3H(Jax) Virgin Mice*

| | Age groups | | | |
|---|---|---|---|---|
| Strain | 4 months | 8 months | 12 months | Tumour bearing |
| C3H(Jax) . | 0·19±0·01 | 0·3±0·08 | 0·29±0·05 | 1·93±0·3* |
| C57(Jax) . | 0·29±0·03 | 0·2±0·01 | 0·21±0·03 | — |

Enzyme activity is expressed in terms of μg. of urea formed per hour per μg. of protein.
* Represents statistically significant when compared with the values of 4-month-old group of the same strain.
Values represent mean of six experiments.

TABLE II.—*Activity of Glucose-6-Phosphate Dehydrogenase in Mammary and Tumour Tissue of C3H(Jax) Virgin Mice*

| | Age groups | | | |
|---|---|---|---|---|
| Strain | 4 months | 8 months | 12 months | Tumour bearing |
| C3H(Jax) . | 0·40±0·07 | 0·36±0·01 | 0·42±0·05 | 0·67*±0·02 |
| C57BL . | 0·35±0·05 | 0·32±0·1 | 0·39±0·05 | — |

Enzyme activity is expressed in terms of change in optical density at 360 Å per minute per μg. of protein.
* Statistically significant when compared with the values of 4-month-old group.
Values represent mean of 6 experiments.

Table II shows the activity of glucose-6-phosphate dehydrogenase in the two strains. In both strains the enzyme activity does not differ appreciably up to the age of 12 months. In tumour tissue the enzyme activity rises significantly. In this case too the enzyme activity is comparable in both strains.

TABLE III.—*Activities of Arginase and Glucose-6-Phosphate Dehydrogenase in Normal Mammary Gland, Precancerous Nodule and Mammary Tumour*

| Enzyme | Normal mammary gland | Precancerous nodule | Tumour |
|---|---|---|---|
| Arginase | 0·22±0·01 | 1·4±0·35* | 1·93±0·4* |
| Glucose-6-phosphate dehydrogenase | 0·38±0·03 | 0·6±0·03* | 0·67±0·02* |

Arginase activity is measured in terms of μg. of urea formed per hour per μg. of protein.
Glucose-6-phosphate dehydrogenase activity is measured in terms of change in optical density at 3600 Å per minute per μg. of protein.
Values represent mean of 6 experiments with standard error.
* Statistically significant when compared with the values of normal mammary gland.

Table III shows the activities of arginase and glucose-6-phosphate dehydrogenase in normal mammary gland at the age of 4 months, in precancerous nodule and in mammary tumour in C3H(Jax) strain. It may be noted that precancerous nodule and the tumour have comparable activities of both of these enzymes which are significantly higher than those present in the normal mammary gland.

### DISCUSSION

The presence of arginase activity in mammary tissue has been acknowledged by various workers (Greenstein *et al.*, 1941; Folley and Greenbaum, 1946), but its precise role in the mammary gland is not at present clearly understood. Folley and Greenbaum (1947) have proposed that probably deamination of gluconeogenic amino acids is carried out in the mammary tissue and arginase may then be playing some role in lactose biosynthesis. High arginase activity in mammary tumour has been reported previously (Greenstein 1954; Smith and Richterich, 1957). Similarly increased glucose-6-phosphate dehydrogenase activity in mammary and other types of tumours is also observed previously (Ono *et al.*, 1963; Hershey *et al.*, 1966). The present data support these earlier observations. It is conceivable that the activity of glucose-6-phosphate dehydrogenase, an enzyme involved in nucleic acid biosynthesis, rises considerable to correspond with the increase in cell proliferation and growth.

Whilst the mammary tissue of 8 and 12 months old C3H(Jax) virgin mice does not show any appreciable difference in the activity of either enzyme when compared with those in the mammary tissue of 4-month-old virgin mice of the same strain, the activities of these same two enzymes increase remarkably in the isolated precancerous nodules and are comparable with those in mammary tumour. In our earlier work it was observed that certain parameters such as nucleic acid levels, activities of RNAse and ATPse change appreciably at the age of 8 months when precancerous hyperplastic nodules are observed in the mammary tissue (Sheth *et al.*, 1967). However, in the present case it is possible that the extent of alterations in the activities of these enzymes is not sufficient to show in the homogenate of composite mammary tissue, consisting mainly of normal mammary gland with few precancerous hyperplastic nodules. The changes in the activities of these two enzymes are perceivable only when the nodules are free of normal mammary tissue. A similar type of observation has been reported for hepatoma cell suspensions (Bhide, 1970). Thus the present experiments bring out the importance of studies on isolated precancerous nodules (free of mammary tissue) which may help to locate subtle metabolic changes associated with the transformation of normal to malignant tissue. Studies on these lines are in progress in this laboratory.

The author is grateful to Dr. K. J. Ranadive, Head of the Biology Division, for her encouragement and interest in this project.

### REFERENCES

Archibald, R. M. C.—(1944) *J. biol. Chem.*, **157**, 507.

Bhide, S. V.—(1970) *Br. J. Cancer*, **24**, 869.

Brown, G. W. and Cohen, P. P.—(1959) *J. biol. Chem.*, **234**, 1769.

Folley, S. J.—(1949) *Biol. Rev.*, **24**, 316.

Folley, S. J. and Greenbaum, A. L.—(1946) *Biochem. J.*, **40**, 46.—(1947) *Biochem. J.*, **41**, 261.

Greenstein, J. P.—(1954) ' Biochemistry of Cancer '. New York (Academic Press Inc.).

Greenstein, J. P., Jenrette, W. V., Mider, G. B. and White, J. C.—(1941) *J. natn. Cancer Inst.*, **2**, 293.

Hershey, F. B., Johnson, G., Murphy, S. M. and Smith, M.—(1966) *Cancer Res.*, **26**, 265.

Löhr, G. W. and Waller, H. D.—(1963) ' Methods of Enzymatic Analysis '. Edited by H. U. Bergmeyer. New York (Academic Press Inc.), p. 744.

Lowry, O. H., Rosenbourgh, N. J., Farr, A. L. and Randall, R. J.—(1951) *J. biol. Chem.*, **193**, 265.

Ono, T., Potter, V. R., Pitot, H. C. and Morris, H. P.—(1963) *Cancer Res.*, **23**, 385.

Sheth, N. A., Bhide, S. V. and Ranadive, K. J.—(1968) *Br. J. Cancer*, **22**, 833.—(1970) *Indian J. Cancer* (in press).

Sheth, N. A., Wagle, M. M., Bhide, S. V. and Ranadive, K. J.—(1967) *Br. J. Cancer*, **21**, 221.

Smith, T. C. and Richterich, B.—(1957) *Cancer Res.*, **17**, 1006.